# Diagnostic Thresholds for Pre–Diabetes Mellitus and Diabetes Mellitus and Subclinical Cardiac Disease in the General Population: *Data From the ACE 1950 Study*

**DOI:** 10.1161/JAHA.120.020447

**Published:** 2021-05-17

**Authors:** Peder L. Myhre, Magnus N. Lyngbakken, Trygve Berge, Ragnhild Røysland, Erika N. Aagaard, Osman Pervez, Brede Kvisvik, Jon Brynildsen, Jon Norseth, Arnljot Tveit, Kjetil Steine, Torbjørn Omland, Helge Røsjø

**Affiliations:** ^1^ Department of Cardiology Akershus University Hospital Lørenskog Norway; ^2^ Department of Multidisciplinary Laboratory Medicine and Medical Biochemistry Akershus University Hospital Lørenskog Norway; ^3^ Division for Research and Innovation Akershus University Hospital Lørenskog Norway; ^4^ Institute for Clinical Medicine University of Oslo Norway; ^5^ Department of Medical Research Vestre Viken Hospital Trust Bærum Norway; ^6^ Department of Laboratory Medicine Vestre Viken Hospital Trust Bærum Norway

**Keywords:** biomarker, diabetes mellitus, echocardiography, hemoglobin A1c, troponin, Biomarkers, Cardiovascular Disease, Echocardiography, Risk Factors

## Abstract

**Background:**

Diabetes mellitus (DM) is associated with left ventricular remodeling and incident heart failure, but the association between glycated hemoglobin A1c (HbA1c) and subclinical cardiac disease is not established. We aimed to determine the associations between HbA1c and (1) echocardiographic measures of left ventricular structure and function, and (2) cardiovascular biomarkers: cardiac troponin T, NT‐proBNP (N‐terminal pro‐B‐type natriuretic peptide), and CRP (C‐reactive protein).

**Methods and Results:**

Participants (n=3688) born in 1950 from the population‐based ACE (Akershus Cardiac Examination) 1950 Study were classified as DM (HbA1c≥6.5% or self‐reported DM), pre‐DM (HbA1c 5.7%–6.5%), and no‐DM (HbA1c<5.7%). DM, pre‐DM, and no‐DM were classified in 380 (10%), 1630 (44%), and 1678 (46%) participants, respectively. Mean age was 63.9±0.7 years, mean body mass index was 27.2±4.4 kg/m^2^, and 49% were women. Higher HbA1c was associated with worse left ventricular systolic (ejection fraction and global longitudinal strain) and diastolic (E/e'‐ratio) function, myocardial injury (cardiac troponin T), inflammation (CRP), and impaired neurohormonal homeostasis (NT‐proBNP) (*P*<0.001 in unadjusted and *P*<0.01 in adjusted analysis for all). The associations between HbA1c and cardiovascular biomarkers were independent of the echocardiographic variables, and vice versa. Associations were nonlinear (*P*<0.05 for nonlinearity) and appeared stronger in the pre‐DM range of HbA1c than the no‐DM and DM range.

**Conclusions:**

HbA1c was associated with indexes of subclinical cardiovascular disease, and this was more pronounced in pre‐DM. Our results suggest that cardiovascular preventive measures should be considered also in subjects with hyperglycemia and HbA1c below the established DM cutoff.

**Registration:**

clinicaltrials.gov. Identifier: NCT01555411.

**Nonstandard Abbreviations and Acronyms**
cTnT: cardiac troponin T
DM: diabetes mellitus
GLS: global longitudinal strain
TRVmax: tricuspid regurgitation velocity maximum jet velocity

**Clinical Perspective**

**What Is New?**

- Diagnostic thresholds for pre–diabetes mellitus (DM) and DM are based on microvascular disease progression, whereas the association with subclinical cardiac disease is unknown.
- We found independent associations between increasing hemoglobin A1c and 6 markers of cardiac dysfunction: cardiac troponin T, C‐reactive protein, N‐terminal pro‐B‐type natriuretic peptide, global longitudinal strain, left ventricular ejection fraction, and E/e'‐ratio.
- These relationships were nonlinear and most pronounced in the pre‐DM range, with weaker associations in the no‐DM and DM range.

​

**What Are the Clinical Implications?**

- Higher concentrations of hemoglobin A1c are associated with worse left ventricular systolic and diastolic function, cardiac injury, inflammation, and impaired neurohormonal regulation.
- Preventive measures for cardiovascular disease should also be considered in patients with hyperglycemia and hemoglobin A1c below the established cutoff for DM.

​

​

The prevalence of diabetes mellitus (DM) is rising drastically worldwide, and according to the most recent report from Global Burden of Disease, it increased from approximately 333 million people in 2005 to 478 million people in 2017.^1^ Pre‐DM is even more common and is estimated to have twice the prevalence of DM.^2^ Although the incidence of cardiovascular events among subjects with DM has been declining over the past 2 decades because of improvements in DM care, there is still a substantial residual risk associated with DM compared with age‐ and sex‐matched controls.^3^ Nonetheless, people with DM who are free of other risk factors seem to have little or no excess risk of myocardial infarction, stroke, and mortality.^4^ This is in contrast to the risk for heart failure (HF), which is substantially elevated in DM, irrespective of the presence of other risk factors.^4^ Indeed, the risk of HF increases with the degree of glycemia in people with established DM,^5^ but also among subjects free of DM.^6^, ^7^ The association of glycated hemoglobin A1c (HbA1c) with HF risk seems to be independent of hypertension, obesity, and other traditional risk factors, suggesting a direct effect of hyperglycemia on HF development.^6^

Progressive changes in cardiac structure and function, also before the development of symptoms, represent the early (subclinical) phase of HF development.^8^ People with DM have adverse cardiac structure and function, also after adjusting for established cardiovascular risk factors.^9^ Worsening glucose tolerance and insulin resistance are associated with increased left ventricular (LV) mass.^10^ Moreover, pre‐DM and duration of DM are associated with subclinical alterations in LV structure and systolic function, which suggest that there may be a continuum of cardiac remodeling across the glycemic spectrum.^11^, ^12^ Still, limited information is available on glycemic thresholds for pre‐DM and DM and subclinical cardiac disease in midlife, a period where many people are at risk of progressing from subclinical disease to symptomatic stages.^8^ Such studies will require detailed cardiac imaging in a large number of participants from the general population. Recent publications from community‐derived cohorts also demonstrate that cardiac biomarker measurements provide additional information to imaging for assessing the risk of HF development.^13^, ^14^ Accordingly, we hypothesized that chronic hyperglycemia is associated with echocardiographic and biochemical indexes of subclinical cardiac injury and dysfunction, in middle‐aged subjects from the general population.

## METHODS

The data set used in this study is not publicly available; the Data Protection Authority approval and patient consent do not allow for such publication. However, the study group welcomes initiatives for cooperation, and data access may be granted on application. More information is available on the study website (http://www.ace1950.no).

### Study Design and Population

The ACE (Akershus Cardiac Examination) 1950 Study is an ongoing epidemiologic cohort study that recruited 3706 men and women born in 1950 from Akershus County, Norway (64% of all alive residents born that year). The baseline visit, which makes the basis for the current analysis, was conducted in 2012 to 2015 at 2 field centers: Akershus University Hospital and Bærum Hospital, Vestre Viken Hospital Trust. Details of the study design and methods have previously been published.^15^ The study protocol was approved by the Regional Ethics Committee (2011/1475) and the local institutional review boards, and all participants provided written informed consent.

For the present analysis, we excluded 18 participants with missing measurements of HbA1c, cardiac troponin T (cTnT), CRP (C‐reactive protein), or NT‐proBNP (N‐terminal pro‐B‐type natriuretic peptide), leaving a study cohort of 3688 participants. We classified participants according to the recommendations from the American Diabetes Association 2019 Guidelines^16^ as DM (HbA1c≥6.5% *or* self‐reported DM *or* use of antidiabetic medication), pre‐DM (HbA1c 5.7%–6.4% and no self‐reported DM *or* use of antidiabetic medication), and no‐DM (HbA1c<5.7% and no self‐reported DM *or* use of antidiabetic medication).

### Blood Sampling and Laboratory Measurements

HbA1c, glucose, CRP, creatinine, cTnT, and NT‐proBNP were measured from blood samples drawn from participants at the baseline visit. HbA1c was measured immediately in EDTA whole blood, and glucose, CRP, and creatinine in serum, at the central routine laboratory at each hospital. Estimated glomerular filtration rate was calculated by the Chronic Kidney Disease Epidemiology Collaboration formula.^17^ cTnT and NT‐proBNP were measured from stored serum samples (−80°C) that were thawed and analyzed in a single batch. cTnT was measured using the troponin T high‐sensitivity STAT assay (Roche Diagnostics, Basel, Switzerland), with 3 ng/L as the limit of detection. NT‐proBNP was measured with the proBNP assay (Roche Diagnostics), with 5 ng/L as the limit of detection. CRP was measured by the CRP (Latex) High Sensitive Immunoturbidimetric assay (Roche Diagnostics), with variation in limit of detection between the study centers and during the inclusion period (<1.0 mg/L, <2.9 mg/L, and <3.0 mg/L). Participants with a concentration below the detection limit were assigned half the limit of detection for all assays.

### Echocardiography

Comprehensive 2‐dimensional Doppler, tissue Doppler imaging, and speckle‐tracking echocardiography were performed at the baseline visit using GE Vivid E9 with the M5S probe, and stored in EchoPAC 201 (GE Healthcare, Horten, Norway) for subsequent off‐line analysis, as previously described.^18^ Quantitative measures were performed according to recommendations from the European Association of Cardiovascular Imaging and American Society of Echocardiography.^19^, ^20^ LV mass was calculated from M‐mode measurements in parasternal long‐axis view, and indexed to body surface area. The ratio between early diastolic velocity and the average of septal and lateral peak early diastolic velocity by tissue Doppler (E/e'‐ratio) was calculated to assess diastolic function. LV ejection fraction (LVEF) was calculated using the modified Simpson biplane method. Global longitudinal strain (GLS) was analyzed semiautomatically by myocardial tracing in the 3 apical views using a 17‐segment model in participants with sufficient image quality to perform the analysis (n=2531; 69%). GLS represents an index of deformation and is reported on a negative scale for myocardial segments that contract (ie, higher [less negative] values refer to worse systolic function [less deformation]). For the statistical analyses, we used GLS as absolute values (ie, higher values equaling better LV function). Estimated systolic pulmonary arterial pressure was assessed by the tricuspid regurgitation maximum jet velocity (TRVmax).

### Statistical Analysis

Participants were categorized on the basis of DM status, and clinical characteristics, echocardiographic variables, and cardiac biomarker concentrations were compared for trend, using linear or logistic regression, across the 3 groups (DM, pre‐DM, and no‐DM). For echocardiographic parameters and cardiac biomarkers, further adjustments were made for clinical variables selected a priori: age, sex, body mass index (BMI), smoking status, prevalent hypertension, atrial fibrillation, coronary artery disease, and estimated glomerular filtration rate. To assess independent predictors of HbA1c, we performed multivariable linear regression models with the echocardiographic parameters and cardiovascular biomarkers as dependent variables. The regression analyses for echocardiographic variables were adjusted for cTnT, NT‐proBNP, and CRP, in addition to the clinical variables described above. The biomarker regression analysis was adjusted for key echocardiographic measures of LV structure, systolic function, and diastolic function: LV mass index, LVEF, and E/e', in addition to the clinical variables. There was substantial missing data for GLS and TRVmax, and high degree of collinearity between GLS and TRVmax and the 3 echocardiographic variables already included. Hence, GLS and TRVmax were adjusted for in additional sensitivity analyses. We did not adjust for multiple testing. We performed interaction analysis for sex and DM category to adjust for effect modification. Univariable and multivariable logistic regression analyses were also conducted with outcome variables dichotomized at the median (ie, 6 ng/L for cTnT, 55 ng/L for NT‐proBNP, 1.5 mg/L for CRP, 55.5% for LVEF, 20.2% for GLS, and 8.6 for E/e'). We used restricted cubic splines to assess the nonlinear association between HbA1c and each biomarker concentration. We used the lowest Akaike Information Criterion to determine the number of knots with the best model fit. We did not force the position of the knots or adjust for possible confounders to assess the natural association between each biomarker concentration and HbA1c. We performed sensitivity analysis in participants free of self‐reported HF and by replacing BMI with waist‐hip ratio. Statistical analysis was performed using STATA software v15.1 (StatCorp, College Station, TX). A 2‐sided *P* value <0.05 was considered significant.

## RESULTS

### Baseline Characteristics

Among the 3688 participants included, 1795 (49%) were women, mean age was 63.9±0.7 years, and mean BMI was 27.2±4.4 kg/m^2^. DM was present in 380 (10%), pre‐DM in 1630 (44%), and no‐DM in 1678 (46%) participants. Of the participants with DM, 265 (70%) self‐reported established DM, of which 20 (8% of the DM population) were type 1 DM, 238 (90%) were type 2 DM, and 7 (2%) were not classified. Two hundred of the participants with DM used antidiabetic medication (53% of the DM population), with 160 participants using noninsulin medications, 23 participants using insulin, and 17 participants using a combination of insulin and other antidiabetic medication. Mean HbA1c in total population was 5.8±0.7% (range, 3.8%–12.7%), and mean fasting glucose was 5.5±1.2 mmol/L (range, 3.6–18.8 mmol/L).

Participants in higher glycemic categories were more likely to be men, to be obese, and to have preexisting hypertension, hypercholesterolemia, coronary artery disease, and HF. These participants were more frequently using cardioprotective and antidiabetic therapy and had lower cholesterol concentrations (Table 1).

**Table 1:** Baseline Characteristics of the Study Population, Stratified by DM Category

Characteristics	No‐DM	Pre‐DM	DM	*P* for trend
	(n=1678)	(n=1630)	(n=380)	
HbA1c, range/mean±SD, %	3.8–5.6/5.4±0.2	5.7–6.4/5.9±0.2	5.1–12.7/7.1±1.1	
Age, y	64.0±0.6	63.8±0.7	63.9±0.7	<0.001
Male sex	855 (51.0)	781 (47.9)	257 (67.6)	<0.001
Current smoker	210 (12.6)	257 (15.9)	63 (16.7)	0.15
Waist‐hip ratio	0.90±0.09	0.92±0.09	0.99±0.09	<0.001
Body mass index, kg/m^2^	26.3±3.9	27.3±4.3	30.4±5.2	<0.001
Obesity	269 (16.0)	370 (22.7)	192 (50.5)	<0.001
Hypertension	948 (56.5)	1028 (63.1)	308 (81.1)	<0.001
Hypercholesterolemia	774 (46.2)	915 (56.3)	246 (65.3)	<0.001
Atrial fibrillation	70 (4.2)	75 (4.6)	20 (5.3)	0.33
Coronary artery disease	71 (4.2)	136 (8.3)	56 (14.7)	<0.001
Heart failure	11 (0.7)	34 (2.1)	15 (3.9)	<0.001
Antihypertensives	7 (0.4)	7 (0.4)	6 (1.6)	0.040
Diuretics	43 (2.6)	45 (2.8)	24 (6.3)	0.003
β Blockers	166 (9.9)	225 (13.8)	103 (27.1)	<0.001
Calcium antagonists	93 (5.5)	131 (8.0)	75 (19.7)	<0.001
RAAS inhibitors	342 (20.4)	467 (28.7)	182 (47.9)	<0.001
Statins	314 (18.7)	446 (27.4)	203 (53.4)	<0.001
Insulin	0 (0.0)	0 (0.0)	40 (10.5)	<0.001
Noninsulin antidiabetics	0 (0.0)	0 (0.0)	177 (46.6)	<0.001
Hemoglobin, g/L	14.3 (13.5–15.0)	14.2 (13.5–15.0)	14.4 (13.6–15.3)	0.27
Total cholesterol, mmol/L	5.5 (4.8–6.2)	5.5 (4.7–6.2)	4.9 (4.0–5.6)	<0.001
LDL cholesterol, mmol/L	3.3 (2.7–3.9)	3.4 (2.6–4.0)	2.8 (2.0–3.5)	<0.001
Triglycerides, mmol/L	1.1 (0.8–1.5)	1.2 (0.9–1.7)	1.6 (1.2–2.3)	<0.001
Creatinine, µmol/L	75.0 (66.0–85.0)	74.0 (66.0–84.0)	75.0 (66.0–87.0)	0.62
eGFR, mL/min per 1.73 m^2^	85.1 (75.5–92.4)	84.9 (75.1–92.5)	88.3 (74.8–94.9)	0.07

Continuous variables are presented as mean±SD and median (quartile 1–quartile 3). Other variables are given as number (percentage). DM indicates diabetes mellitus; eGFR, estimated glomerular filtration rate; HbA1c, hemoglobin A1c; LDL, low‐density lipoprotein; and RAAS, renin‐angiotensin‐aldosterone system.

### Echocardiographic Variables and Cardiac Biomarkers Across the Glycemic Spectrum

After adjusting for clinical characteristics, higher categories of DM (no‐DM, pre‐DM, and DM) were associated with worse LV systolic function (lower LVEF [*P*=0.002] and GLS [*P*<0.001]), worse LV diastolic function (higher E/e'‐ratio [*P*<0.001]), and higher concentrations of cTnT (*P*<0.001), NT‐proBNP (*P*<0.001), and CRP (*P*<0.001) (Table S1). There was no significant association between HbA1c and LV mass index (*P*=0.23) or TRVmax (*P*=0.09) in adjusted models.

To assess independent associations of HbA1c with echocardiographic variables and cardiac biomarkers, we included these in the same model (ie, echocardiographic variables adjusted for cardiac biomarkers and vice versa). Higher HbA1c remained associated with lower LVEF (*P*=0.009), worse GLS (*P*<0.001), and higher E/e'‐ratio (*P*=0.001) after adjusting for clinical characteristics and cTnT, NT‐proBNP, and CRP (Table 2). Higher HbA1c also remained associated with higher cTnT (*P*<0.001), higher CRP (*P*<0.001), and lower NT‐proBNP (*P*<0.001) concentrations, after adjusting for clinical characteristics and LV mass index, LVEF, and E/e' (Table 2). These associations persisted after additional adjustments for GLS and TRVmax among participants with these measures available (n=1966; 53%). Overall, the strongest associations to HbA1c in adjusted models were seen for E/e'‐ratio (β, 0.52 [95% CI, 0.38–0.65] per unit increase; t=7.7; *P*<0.001) and NT‐proBNP (β, −0.22 [95% CI, −0.28 to −0.16] per doubling; t=−7.2; *P*<0.001).

**Table 2:** Association of HbA1c (Exposure Variable) With Echocardiographic Parameters and Cardiac Biomarkers (Outcome Variables)

Variable	Coefficient (95% CI)	t	*P* value
Echocardiographic parameters			
LV mass index, g/m^2^	−0.50 (−1.41 to 0.40)	−1.1	0.28
LV ejection fraction, %	−0.40 (0.71 to −0.10)	−2.6	0.009
Average peak global longitudinal strain, %*	0.38 (0.22 to 0.55)	4.6	<0.001
E/e'‐ratio	0.52 (0.38 to 0.65)	7.7	<0.001
Tricuspid regurgitation maximum velocity, m/s^†^	0.01 (−0.00 to 0.03)	1.4	0.14
Cardiac biomarkers			
Cardiac troponin T, per doubling	0.09 (0.05 to 0.14)	4.4	<0.001
NT‐proBNP, per doubling	−0.22 (−0.28 to −0.16)	−7.2	<0.001
CRP, per doubling	0.11 (0.06 to 0.17)	4.3	<0.001

Analyses of echocardiographic parameters are adjusted for cardiac biomarkers, and analyses of cardiac biomarkers are adjusted for echocardiographic parameters, in addition to clinical characteristics for both. Echocardiographic parameters are adjusted for age, sex, body mass index, smoking status, hypertension, atrial fibrillation, coronary artery disease, estimated glomerular filtration rate+cardiac troponin T, NT‐proBNP, and CRP. Cardiac biomarkers are adjusted for age, sex, body mass index, smoking status, hypertension, atrial fibrillation, coronary artery disease, estimated glomerular filtration rate+LV mass index, LV ejection fraction, and E/e'‐ratio. CRP indicates C‐reactive protein; HbA1c, hemoglobin A1c; LV, left ventricular; and NT‐proBNP, N‐terminal pro‐B‐type natriuretic peptide.

* n=2512 with available global longitudinal strain.

^†^ n=2817 with available tricuspid regurgitation maximum velocity.

There were sex differences in strength of the associations between HbA1c and E/e'‐ratio, cTnT, and NT‐proBNP (*P* for interaction <0.001 for all), with stronger associations for cTnT and E/e' with HbA1c in men and stronger associations for NT‐proBNP with HbA1c in women (Figure S1). There was no interaction by sex on the association between HbA1c and LVEF, GLS, and CRP.

LVEF, GLS, E/e'‐ratio, NT‐proBNP, cTnT, and CRP remained associated with HbA1c in a sensitivity analysis that excluded participants with self‐reported HF or LVEF ≤40% (Table S2), and a sensitivity analysis replacing BMI with waist‐hip ratio (Table S3).

### Diagnostic Thresholds for Pre‐DM and DM and Markers of Cardiac Structure, Function, Injury, and Inflammation

**Figure 1:**
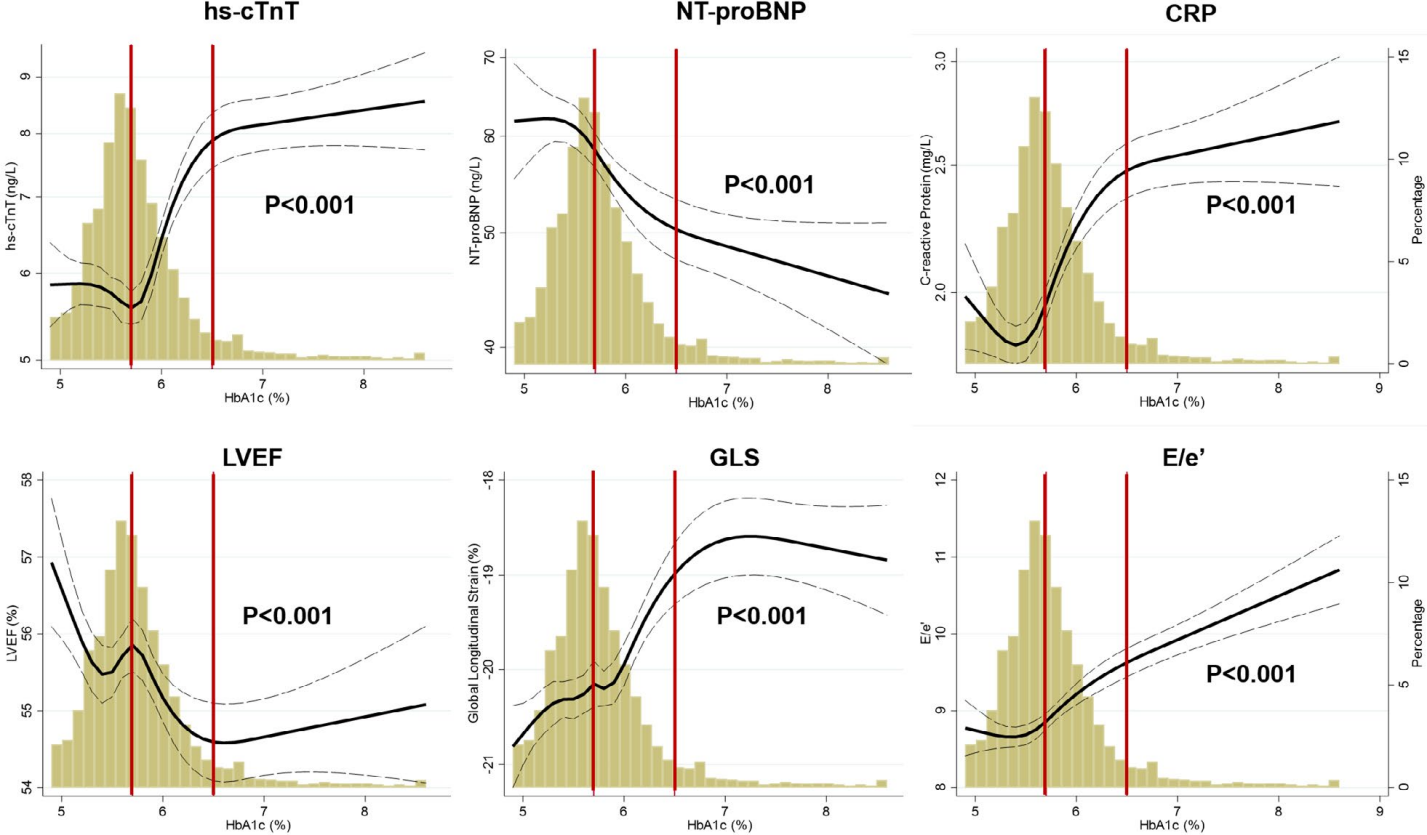
Association between HbA1c and cardiovascular biomarkers independently associated with HbA1c. Fitted restricted cubic splines of log‐transformed hs‐cTnT, NT‐proBNP, CRP, LVEF, global longitudinal strain (GLS), and E/e'‐ratio as a function of HbA1c. The number of knots for each analysis was selected on the basis of the lowest Akaike Information Criterion (6, 4, 4, 5, 7, and 4 knots, respectively). The brown horizontal lines represent the HbA1c threshold for pre–diabetes mellitus (5.7%) and diabetes mellitus (6.5%). The dotted lines reflect the 95% CIs. *P* value is for overall trend. *P* for nonlinearity was <0.001 for all. CRP indicates C‐reactive protein; GLS, global longitudinal strain; HbA1c, hemoglobin A1c; hs‐cTnT, high‐sensitivity cardiac troponin T; LVEF, left ventricular ejection fraction; and NT‐proBNP, N‐terminal pro‐B‐type natriuretic peptide.

**Figure 2:**
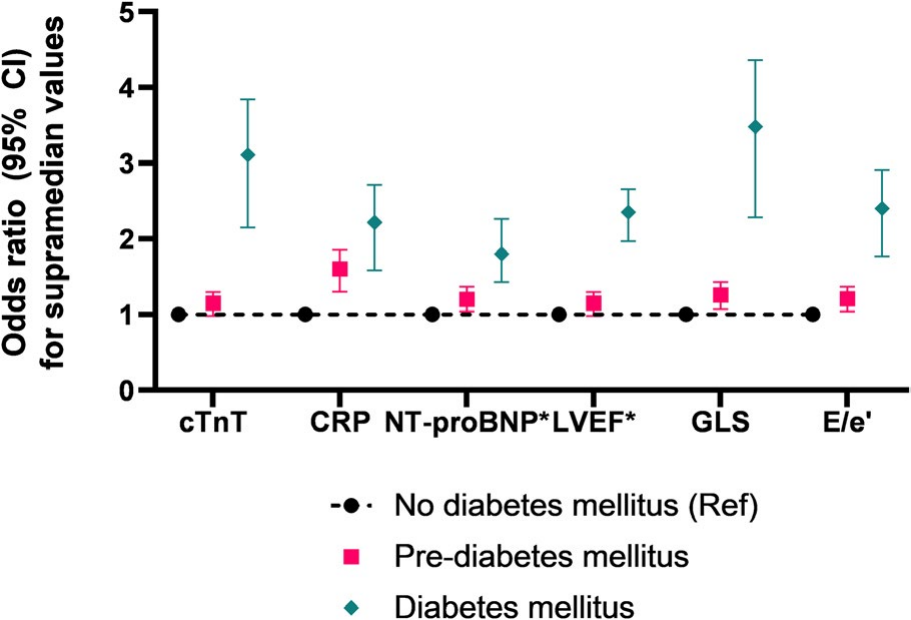
Measures of cardiovascular biomarkers independently associated with hemoglobin A1c in pre–DM and DM, compared with participants free of DM. Odds ratio of log‐transformed cTnT, CRP, GLS, and E/e' ‐ratio above the median and NT‐proBNP and LVEF below the median for participants with pre‐DM and DM, with no‐DM as reference (Ref). Adjusted for age, sex, body mass index, smoking status, prevalent hypertension, atrial fibrillation, coronary artery disease, and estimated glomerular filtration rate. CRP indicates C‐reactive protein; cTnT, cardiac troponin T; DM, diabetes mellitus; GLS, global longitudinal strain; LVEF, left ventricular ejection fraction; and NT‐proBNP, N‐terminal pro‐B‐type natriuretic peptide. *, odds ratio for inframedian values.

There were nonlinear associations between HbA1c and LVEF, GLS, E/e'‐ratio, NT‐proBNP, cTnT, and CRP (*P* for nonlinearity <0.05 for all). The best fit for all 6 associations using restricted cubic spline yielded an S‐shaped association for these associations (≥4 knots for all; Figure 1). There was an interaction by DM category on the association between HbA1c and each of these biomarkers (*P*<0.001 for all), except for LVEF (*P*=0.71) (Table 3). In general, the strongest association was seen in the pre‐DM group for all 6 biomarkers. This is presented in Figure 1 where, in general, the steepest part of the spline curves between HbA1c and each of the variables was in the pre‐DM range (5.7%–6.4%), whereas the curves were flatter in the no‐DM range (<5.7%) and in the DM range (≥6.5%). The exceptions were a significant association between HbA1c and systolic function (LVEF and GLS) in no‐DM, and between HbA1c and diastolic function and inflammation (E/e'‐ratio and CRP) in DM (Table 3). These results were consistent irrespective of antidiabetic treatment or not, for all biomarkers in participants with DM. The adjusted odds ratio (95% CI) for participants with pre‐DM and DM, compared with no‐DM, to have supramedian values of cTnT, CRP, GLS, and E/e'‐ratio, and inframedian values of LVEF and NT‐proBNP, is presented in Figure 2.

**Table 3:** Association Between HbA1c and Log‐Transformed cTnT, CRP, NT‐proBNP, LVEF, GLS, and E/e'‐ratio in Each Glycemia Category by Linear Regression

Variable	cTnT (Log_2_)	CRP (Log_2_)	NT‐proBNP (Log_2_)	LVEF	GLS	E/e'Ratio
Unadjusted						
No‐DM	−0.07 (−0.25 to 0.10), *P*=0.41	−0.05 (−0.25 to 0.15), *P*=0.65	−0.14 (−0.39 to 0.10), *P*=0.25	−1.51 (−2.79 to −0.22), *P*=0.021	0.95 (0.33 to 1.58), *P*=0.003	−0.10 (−0.65 to 0.46), *P*=0.73
Pre‐DM	0.29 (0.09 to 0.50), *P*=0.005	0.71 (0.44 to 0.98), *P*<0.001	−0.27 (−0.56 to 0.03), *P*=0.07	−1.90 (−3.50 to −0.30), *P*=0.02	0.54 (−0.29 to 1.37), *P*=0.20	0.92 (0.24 to 1.59), *P*=0.008
DM	−0.01 (−0.08 to 0.06), *P*=0.84	0.11 (0.00 to 0.21), *P*=0.04	−0.09 (−0.20 to 0.03), *P*=0.15	0.24 (−0.41 to 0.88), *P*=0.47	0.05 (−0.26 to 0.36), *P*=0.77	0.44 (0.16 to 0.73), *P*=0.002
Interaction	*P*<0.001	*P*<0.001	*P*<0.001	*P*=0.71	*P*<0.001	*P*<0.001
Adjusted for age, sex, BMI, smoking status, prevalent hypertension, atrial fibrillation, coronary artery disease, and eGFR						
No‐DM	−0.14 (−0.34 to 0.05), *P*=0.15	−0.04 (−0.25 to 0.16), *P*=0.66	−0.07 (−0.34 to 0.19), *P*=0.59	−1.56 (−2.82 to −0.30), *P*=0.016	0.86 (0.21 to 1.52), *P*=0.01	0.08 (−0.50 to 0.66), *P*=0.80
Pre‐DM	0.54 (0.31 to 0.77), *P*=0<0.001	0.89 (0.62 to 1.16), *P*<0.001	−0.29 (−0.60 to 0.02), *P*=0.07	−1.64 (−3.19 to −0.09), *P*=0.04	1.02 (0.16 to 1.89), *P*=0.02	1.16 (0.46 to 1.85), *P*=0.001
DM	0.04 (−0.04 to 0.13), *P*=0.28	0.11 (0.00 to 0.22), *P*=0.04	−0.06 (−0.19 to 0.06), *P*=0.28	0.26 (−0.37 to 0.89), *P*=0.42	0.14 (−0.18 to 0.46), *P*=0.39	0.48 (0.19 to 0.77), *P*=0.001
Interaction	*P*<0.001	*P*<0.001	*P*<0.001	*P*=0.76	*P*<0.001	*P*<0.001

BMI indicates body mass index; CRP, C‐reactive protein; cTnT, cardiac troponin T; DM, diabetes mellitus; eGFR, estimated glomerular filtration rate; GLS, global longitudinal strain; HbA1c, hemoglobin A1c; LVEF, left ventricular ejection fraction; and NT‐proBNP, N‐terminal pro‐B‐type natriuretic peptide.

Fasting glucose was also associated with LVEF, GLS, E/e', NT‐proBNP, cTnT, and CRP in unadjusted and adjusted models (*P*<0.01 for all). The associations did not have the same S‐shaped association as demonstrated for HbA1c (Figure S2).

## DISCUSSION

Among 3688 participants aged 62 to 65 years from the general Norwegian population, higher HbA1c was independently associated with higher cTnT, CRP, and E/e'‐ratio, and with lower LVEF, GLS, and NT‐proBNP. These relationships were nonlinear and most pronounced in the pre‐DM range of HbA1c, with weaker associations in the no‐DM and DM range. These findings suggest that middle‐aged subjects within the pre‐DM range of HbA1c may have early manifestation of LV dysfunction, injury, inflammation, and neurohormonal dysregulation. Thus, our data support that cardiovascular preventive measures should also be considered in subjects with chronic hyperglycemia below the current diagnostic threshold for DM.

### Subclinical Cardiac Alterations Across the Specter of Glycemia

To our knowledge, this study is the first to assess the association between HbA1c levels and multiple key cardiovascular risk markers, including both echocardiographic and circulating biomarkers, in middle‐aged subjects recruited from the general population. The key novel finding is that worse glycemic status associates with markers of LV systolic dysfunction (LVEF and GLS), LV diastolic dysfunction (E/e'), inflammation (CRP), myocardial injury (cTnT), and impaired neurohormonal homeostasis (NT‐proBNP), and that these associations are *independent* of each other. Notably, all of these markers are associated with increased risk of HF development,^21^, ^22^, ^23^, ^24^, ^25^ and thus seem to represent important pathological pathways linking dysglycemia to HF.

The association between HbA1c and measures of cardiac function and structure has previously been investigated in elderly subjects from the ARIC (Atherosclerosis Risk in Communities) study, where they found GLS, E/e'‐ratio, and LV mass to be the most important echocardiographic correlates of chronic hyperglycemia status.^11^ This is in agreement with our results, except for the association with LV mass, which was non‐significant in our adjusted models. This difference in association with LV structure may relate to the 10‐ to 15‐year younger population in our study or the exclusion of established cardiovascular disease in the ARIC study. ARIC study investigators have also demonstrated a nearly linear association between HbA1c and cTnT in this elderly US population.^26^ CRP is known to associate with hyperglycemia,^27^ and predicts the risk of type 2 DM.^28^ In our study, we extend these findings by demonstrating an independent, and thus additive, link between DM status and LV function, inflammation, and myocardial injury. Furthermore, we demonstrate a robust inverse association between NT‐proBNP and HbA1c, including in models that adjust for other biomarkers and measures of obesity, such as BMI and waist‐hip ratio. BMI is known to correlate inversely with concentrations of natriuretic peptides.^29^ Natriuretic peptides mediate several beneficial cardiovascular effects, such as natriuresis, vasodilatation, lipid mobilization, insulin secretion, and counteracting the renin‐angiotensin‐aldosterone system and sympathetic nervous system.^30^ Low concentrations of natriuretic peptides, also referred to as natriuretic peptide deficiency,^31^ are associated with cardiovascular risk factors, including worse LV systolic function.^32^ The mechanism behind lower NT‐proBNP with increasing HbA1c is unclear, but may be related to adipocyte overexpression of cytokines (the leptin‐neprilysin‐aldosterone axis),^33^ insulin resistance,^34^ or simply impaired neurohormonal hemostasis in obesity.^35^ As an extension to this, our study finds that chronic hyperglycemia is related to low NT‐proBNP *beyond* obesity and measures of cardiac structure, function, injury, and inflammation. NT‐proBNP concentrations above the median (>66 ng/L) were 50% less likely in participants with DM compared with non‐DM.

### Thresholds for Defining Pre‐DM and DM in Association to Markers of Subclinical Cardiovascular Disease

The recently updated American Diabetes Association definition of pre‐DM and DM^16^ recommends an HbA1c threshold of 5.7% and 6.5%, respectively. HbA1c is a frequently used screening test for DM as it reflects average glucose concentrations over the previous 8 to 12 weeks, can be performed at any time of the day, and does not require fasting. The HbA1c thresholds are derived from data on microvascular disease, such as retinopathy and nephropathy, in association with dysglycemia.^36^ As such, the risk of microvascular disease increases sharply around HbA1c levels of 6.5%. Results from our study suggest that the risk of cardiac disease increases earlier than HbA1c of 6.5%; thus, we find evidence of subclinical cardiovascular disease in the pre‐DM range of HbA1c among our participants from the general population. This notion is based on strong associations between HbA1c in the pre‐DM range and echocardiographic and biochemical measures of worsening cardiac function, including cTnT, CRP, NT‐proBNP, LVEF, GLS, and E/e'‐ratio. Finally, around the DM threshold, we found attenuated associations between HbA1c and markers of cardiac dysfunction, with the exception for E/e'‐ratio and NT‐proBNP, that remained associated also in participants with DM. Of note, the lack of statistical significant associations in participants with DM could be a result of limited statistical power to assess this question as we only had 10% of participants fulfilling the criteria for DM.

### Mechanisms Behind HF Risk in DM

Dysglycemia is an independent risk factor for HF, irrespective of DM status.^7^ The link between DM and HF has gained increasing attention in the past few years, partly because of the introduction of sodium‐glucose cotransporter 2 inhibitors, which has consistently been demonstrated to prevent HF development in type 2 DM, despite modest reductions in HbA1c.^37^ Most recently, the sodium‐glucose cotransporter 2 inhibitors dapagliflozin and empagliflozin were proved to markedly reduce risk among patients with HF and reduced LVEF, irrespective of DM status.^38^, ^39^ The mechanisms linking hyperglycemia to HF are complex and not completely understood, but relate to (1) hyperinsulinemia through sodium retention and activation of the sympathetic nervous system,^40^ (2) oxidative stress associated with cell injury or apoptosis, resulting in decreased cardiac contractility,^41^ and (3) production of advanced glycation end products, which induced cardiac fibrosis.^42^ Our study adds to this understanding by demonstrating independent associations of myocardial injury, inflammation, neurohormonal dysregulation, and LV systolic and diastolic dysfunction to chronic hyperglycemia. This emphasizes the complexity of pathological pathways leading to HF development in DM. Prospective, longitudinal studies with serial blood sampling and measures of cardiac structure and function are needed to improve our mechanistic understanding of these pathways.

### Limitations

The number of subjects with DM or undiagnosed DM (HbA1c ≥6.5%) was relatively low, and associations within this group must be interpreted with caution. Missing data on GLS and TRVmax, primarily because of poor image quality,^18^ restricted the sample size in analysis with these variables. However, unmeasureable myocardial strain and TRV in ~60% likely reflect clinical practice. Information on prevalent HF in our population was limited and restricted to self‐report. We excluded these participants in a sensitivity analysis and found consistent associations among participants free of HF. However, we cannot be certain whether a more thorough assessment of HF (ie, by adjudication) would modify the association between HbA1c and the biomarkers measured. The findings in our relatively homogeneous population might not apply to more diverse ethnic populations and populations with higher BMI.

## CONCLUSIONS

HbA1c is nonlinearly, and independently, associated with markers of myocardial injury, inflammation, neurohormonal dysregulation, and LV systolic and diastolic dysfunction, in middle‐aged subjects from the general population. Particularly strong relationships were seen in participants with pre‐DM, which could indicate a need for lifestyle and possibly pharmacological interventions also in subjects with HbA1c levels below the current diagnostic cutoff for DM.

## Sources of Funding

Dr Myhre and Dr Lyngbakken are supported by grants from the South‐Eastern Norway Regional Health Authority. The ACE (Akershus Cardiac Examination) 1950 Study is funded by the 2 health trusts: Akershus University Hospital Trust and Vestre Viken Hospital Trust.

## Disclosures

Dr Myhre has served on advisory boards for Novartis and Novo Nordisk, and has received consulting honoraria from Novartis, AmGen, and Novo Nordisk. Dr Omland has served on advisory boards for Abbott Diagnostics, Roche Diagnostics, and Bayer, and has received research support from Abbott Diagnostics, Novartis, Roche Diagnostics, Singulex, and SomaLogic via Akershus University Hospital, and speaker's or consulting honoraria from Roche Diagnostics, Siemens Healthineers, and CardiNor. Dr Røsjø has received personal fees from Novartis and Thermo Fischer BRAMS, CardiNor, and SpinChip. The remaining authors have no disclosures to report.

## Supporting information

Tables S1–S3
Figures S1–S2
