# Supplementary material for: Diagnostic Thresholds for Pre–Diabetes Mellitus and Diabetes Mellitus and Subclinical Cardiac Disease in the General Population: Data From the ACE 1950 Study
Source: J Am Heart Assoc. 2021 May 17;10(11):e020447. doi: 10.1161/JAHA.120.020447 (PMC8483542; doi:10.1161/JAHA.120.020447)
Supplement: Supplementary file 1 — Tables S1–S3 Figures S1–S2 [file JAH3-10-e020447-s001.pdf]

SUPPLEMENTAL MATERIAL

Table S1. Measures of left ventricular structure and function by echocardiography and cardiac biomarkers stratified by diabetes category, unadjusted and adjusted for age, sex, smoking, body mass index, hypertension, atrial fibrillation, coronary artery disease and estimated glomerular filtration rate.

	No DM n=1678	Pre-DM n=1630	DM n=380	Unadjusted P for trend	Adjusted P for trend
LV mass index (g/m^2)	76.6 ± 18.3	75.8 ± 17.9	80.4 ± 20.6	0.06	0.23
LV ejection fraction (%)	55.8 ± 5.5	55.4 ± 6.0	54.7 ± 6.8	0.003	0.002
Average peak global longitudinal strain (%)	-20.3 ± 2.4	-20.1 ± 2.7	-18.9 ± 2.6	<0.001	<0.001
E/e'	8.7 ± 2.4	9.1 ± 2.6	10.1 ± 3.2	<0.001	<0.001
Tricuspid regurgitation maximum velocity (m/s)	2.23 ± 0.24	2.23 ± 0.27	2.28 ± 0.28	0.017	0.09
Cardiac troponin T (ng/L)	6.0 [4.0 , 8.0]	6.0 [4.9 , 9.0]	8.0 [6.0 , 12.0	<0.001	<0.001
N-terminal pro-B-type natriuretic peptide (ng/L)	59.2 [37.0 , 100.0]	53.5 [33.8 , 91.0]	44.0 [25.4 , 81.0]	<0.001	<0.001
C-reactive protein (ng/L)	1.5 [1.5 , 1.5]	1.5 [1.5 , 1.5]	1.5 [1.5 , 4.0]	<0.001	<0.001

LV = left ventricular; DM = Diabetes mellitus

Continuous variables are presented as mean ± standard deviation and median [quartile 1, quartile 3].

Table S2. Multivariable regression analysis of left ventricular ejection fraction, global longitudinal strain, E/e’, cardiac troponin T, N-terminal pro-B-type natriuretic peptide and C-reactive protein in association with one unit increase in hemoglobin A1c concentrations after excluding n=107 patients with self-reported heart failure or left ventricular ejection fraction ≤40%.

	Coef.	95% Conf. Interval		t	P-value
Left ventricular ejection fraction	-0.40	-0.70	-0.09	-2.6	0.010
Average peak global longitudinal strain	0.38	0.22	0.55	4.5	<0.001
E/e'	0.47	0.34	0.61	7.1	<0.001
Cardiac troponin T	0.09	0.05	0.13	4.3	<0.001
N-terminal pro-B-type natriuretic peptide	-0.23	-0.29	-0.17	-7.5	<0.001
C-reactive protein	0.11	0.06	0.17	4.1	<0.001

Adjusted for age, sex, body mass index, smoking status, hypertension, atrial fibrillation, coronary artery disease, estimated glomerular filtration rate
+ cardiac troponin T, N-terminal pro-B-type natriuretic peptide and C-reactive protein for echocardiographic variables
+ LV mass index, LV ejection fraction and E/e’ for cardiac biomarkers

Table S3. Multivariable regression analysis of left ventricular ejection fraction, global longitudinal strain, E/e', cardiac troponin T, N-terminal pro-B-type natriuretic peptide and C-reactive protein in association with one unit increase in hemoglobin A1c concentrations after replacing body mass index with waist-hip ratio.

	**Coef.**	**95% Conf. Interval**		**t**	**P-value**
Left ventricular ejection fraction	-0.36	-0.67	-0.05	-2.3	0.021
Average peak global longitudinal strain	0.33	0.16	0.49	3.9	<0.001
E/e'	0.47	0.34	0.61	7.1	<0.001
Cardiac troponin T	0.10	0.05	0.14	4.5	<0.001
N-terminal pro-B-type natriuretic peptide	-0.22	-0.28	-0.16	-7.1	<0.001
C-reactive protein	0.12	0.07	0.18	4.5	<0.001

Adjusted for age, sex, body mass index, smoking status, hypertension, atrial fibrillation, coronary artery disease, estimated glomerular filtration rate
+ cardiac troponin T, N-terminal pro-B-type natriuretic peptide and C-reactive protein for echocardiographic variables
+ LV mass index, LV ejection fraction and E/e' for cardiac biomarkers

Figure S1. Fitted restricted cubic splines of log-transformed cardiac troponin T (cTnT), N-terminal pro-B-type natriuretic peptide (NT-proBNP) and E/e’ as a function of glycated hemoglobin A1c for men and women.

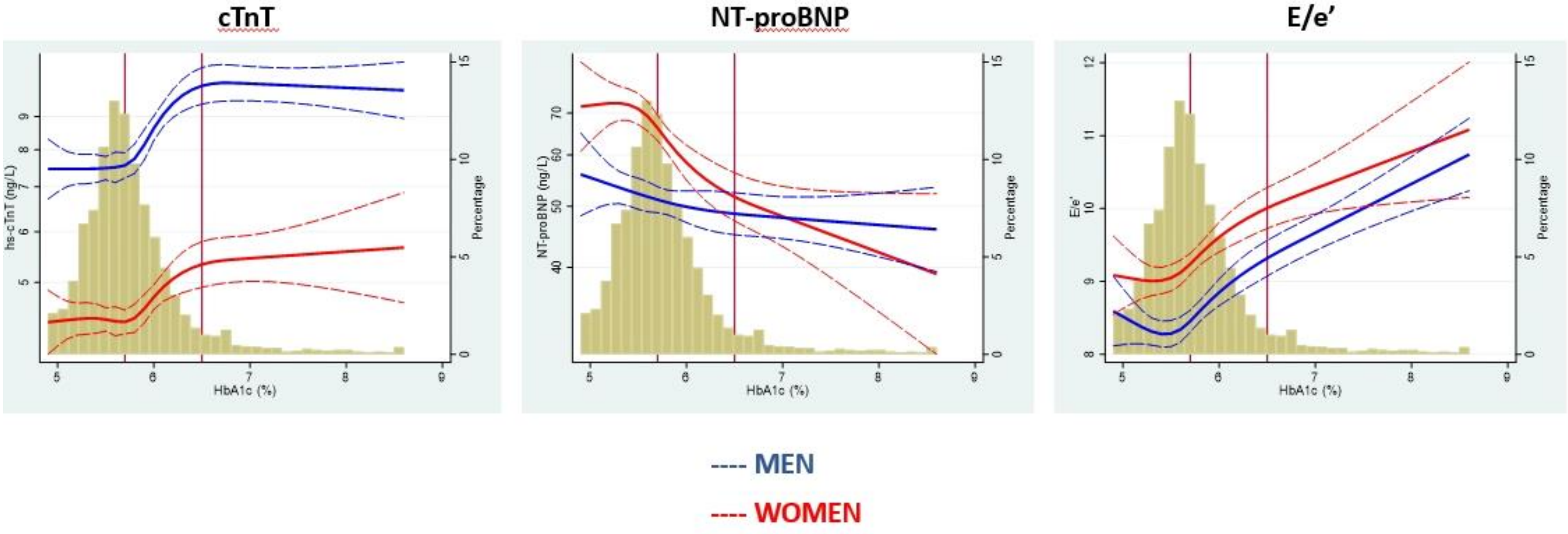

The number of knots for each analysis was selected based on the lowest Akaike Information Criterion. The brown horizontal lines represent the HbA1c threshold for pre-diabetes mellitus (5.7%) and diabetes mellitus (6.5%). The dotted lines reflect the 95% confidence intervals

Figure S2. Fitted restricted cubic splines of log-transformed high-sensitivity troponin T (hs-cTnT), N-terminal pro-B-type natriuretic peptide (NT-proBNP), C-reactive protein (CRP), left ventricular ejection fraction (LVEF), global longitudinal strain (GLS) and E/e’ as a function of fasting glucose.

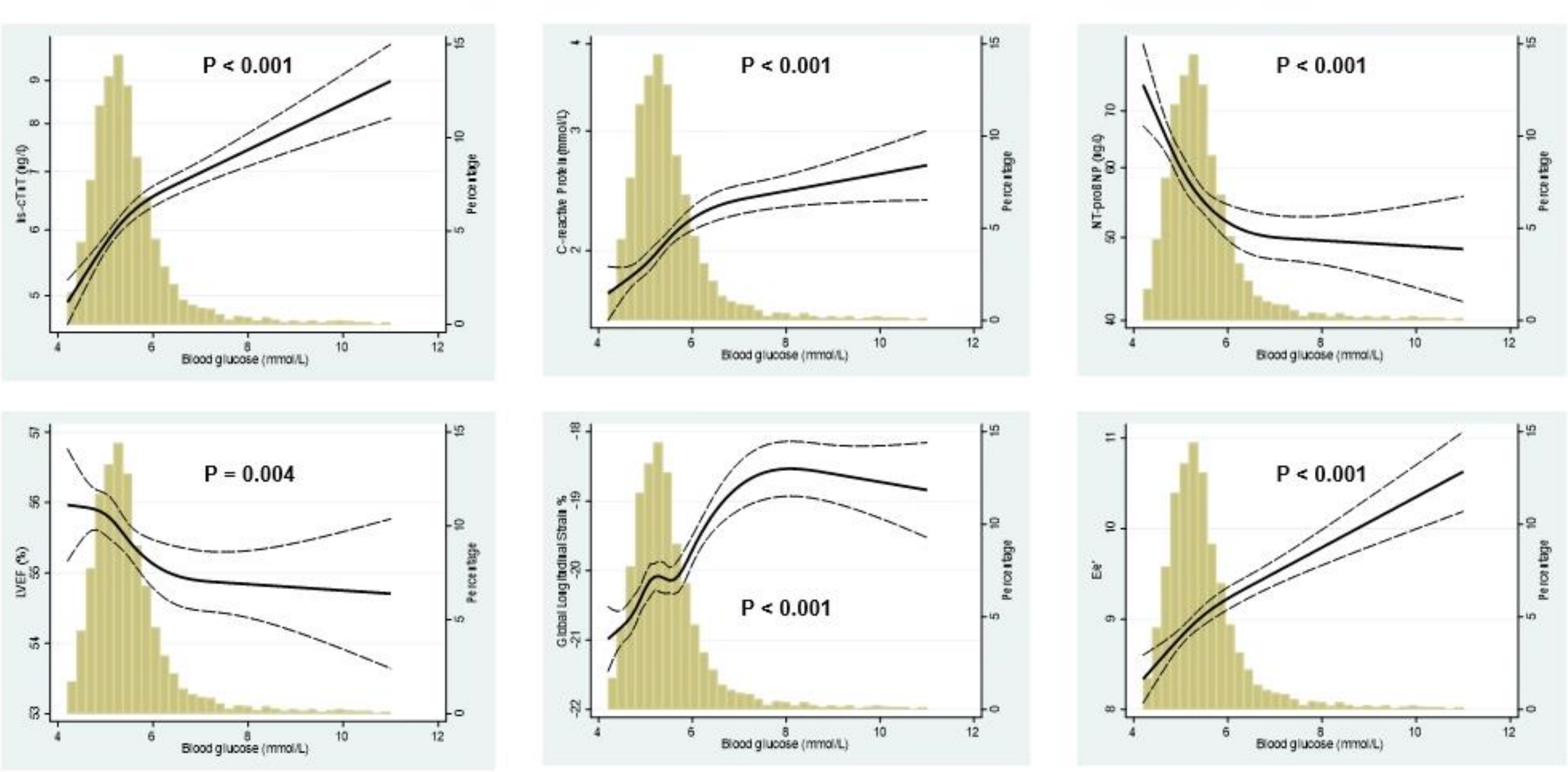

The number of knots for each analysis was selected based on the lowest Akaike Information Criterion. The dotted lines reflect the 95% confidence intervals
